# A Review on Role of Microbiome in Obesity and Antiobesity Properties of Probiotic Supplements

**DOI:** 10.1155/2019/3291367

**Published:** 2019-05-09

**Authors:** Bhagavathi Sundaram Sivamaruthi, Periyanaina Kesika, Natarajan Suganthy, Chaiyavat Chaiyasut

**Affiliations:** ^1^Innovation Center for Holistic Health, Nutraceuticals, and Cosmeceuticals, Faculty of Pharmacy, Chiang Mai University, Chiang Mai- 50200, Thailand; ^2^Department of Nanoscience and Technology, Alagappa University, Karaikudi, India

## Abstract

Probiotics are now recognized for several health benefits and they have been recommended as a complementary therapeutic agent for metabolic disorders. Obesity is an altered health condition, which is a resultant of irregular energy intake and energy balance, changes in gut microbiota, and improper diet with the influence of genetic makeup and environmental factors. Several studies revealed the influence of probiotic supplementation on obesity-associated consequences* in vitro*,* in vivo*, and in human clinical studies. The current manuscript discussed the factors influencing the occurrence of obesity, the interplay between microbiome and obesity, the effect of the probiotic intervention on the health status of obese people, and possible mechanism of antiobesity activity of probiotics. The literature survey revealed that the antiobese activity of probiotics might be associated with their ability to alter the intestinal microbiota, remodeling of energy metabolism, alter the expression of genes related to thermogenesis, glucose metabolism, and lipid metabolism, and change the parasympathetic nerve activity. Further intense research is necessary to figure out the best probiotic or synbiotic mixture and optimum dosage and duration of the intervention to reduce obesity and prevent the recurring of obese condition.

## 1. Introduction

Food habits and lifestyle greatly influence the quality of the life and health status of humans. The improper diet and lifestyle are associated with several metabolic disorders and are the greatest global health issues [1]. The environmental factors, maternal health, and host genetic makeup are also involved in the development of metabolic disorders and diseases. The composition of gut microbiota (GM) and its function is altered due to the consumption of improper diet, which affects the health status of the host, specifically associated with the development of obesity. GM is involved in the energy balancing, intestinal integrity, and immunity against invading pathogens; thereby GM controls the overall health status of the host [2–4].

GM can be positively altered by the supplementation of probiotics, a group of beneficial microbes that confers health benefits when consumed in an adequate amount [5]. Probiotic intervention has been recognized for the treatment or betterment of several ill-health conditions such as diarrhea, allergy, gastrointestinal disorders, and metabolic syndromes [6–11]. The probiotic supplementation also slows down the aging-associated health issues by positive regulation of GM [12, 13].

Obesity is an altered health condition, which is a resultant of irregular energy intake and energy balance, changes in GM, and improper diet with the influence of genetic makeup and environmental factors. Obesity is defined as an accumulation of excessive fat that impairs health status [14]. The obesity rate is drastically increased over the last decades [15], and it has been estimated that about 1.9 billion adults were overweight and, among them, 650 million adults are obese in 2016 [16]. The overweight with some ill-health conditions such as diabetes, hypertension, and cardiovascular diseases is considered as morbid obesity [14, 17].

GM is one of the influencing environmental factors involved in the initiation of obesity mainly by disturbing the food intake and energy balance. Though some of the* in vivo* studies demonstrated the role of GM in the onset of obesity, the exact etiology of obesity has not yet been explained [2, 14, 18, 19]. As mentioned earlier, probiotic is a feasible way to regulate and improve the GM. In recent decades, several studies have focused on the influence of probiotic supplementation on the health improvement of obese people, and the results were found to be controversial. The current manuscript summarizes and discusses the outcome of clinical trials conducted to evaluate the probiotic based supplementation on the health status of obese people. The published scientific documents have been searched and retrieved from Scopus, Web of Science, PubMed, and Google Scholar using the search terms “Probiotic” and “obesity.” The relevant scientific documents in English have been selected without any chronological restrictions for the preparation of the current manuscript.

## 2. Obesity: Influencing Factors and Consequences

Several factors are associated with the development of obesity. Use of high-calorie fast-foods, high consumption rate, less physical powered occupations, lack of physical activities, insufficient sleep, side effects from the medicines like topiramate, olanzapine, and pioglitazone, and other environmental, genetic, and socioeconomic factors are closely related to the onset of obesity [20–23].

Energy imbalance, environmental factors, and genetic makeup are significantly connected to a network that regulates several physiological functions. The neuronal system regulates the energy expenditure through the stimulants from the gastrointestinal tract in the form of neurotransmitters and other neuropeptides produced by GM. The regulatory molecules released by the microbiota influence the brain regions, which is responsible for cognitive functions, emotions, and food consumption. The negative energy balance (due to increased physical activity or reduced food consumption or both) plays a vital role in obesity in association with energy expenditure, physical and metabolic activities, and orexigenic signals [55–59].

**Figure 1:**
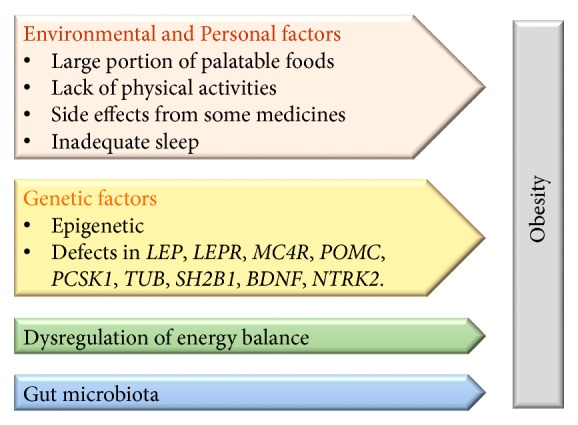
The factors influencing the incidence and development of obesity.

Pigeyre et al. [59] reviewed the genes associated with monogenic obesity in humans. The mutation in leptin (leptin is an adipocyte-specific secreted protein associated with energy expenditure and appetite), leptin receptor, melanocortin 4 receptor (a G-protein-coupled receptor implicated in energy homeostasis), and prohormone convertase 1 (involved in managing prohormones), defects in proopiomelanocortin precursor (precursor of adrenocorticotrophin, melanocyte-stimulating hormones, and opioid-receptor ligand beta-endorphin), tyrosine receptor kinase B (neurotrophic receptor expressed in neuronal and nonneuronal tissues and associated with several physiological regulations and processes such as synaptic plasticity and hyperphagia), brain-derived neurotrophic factor (involved in neuronal plasticity and cognitive function and acting as modulator of neurotransmitter), kinase suppressor of Ras 2 (molecular scaffold expressing majorly in brain), and tubby bipartite transcription factor were associated with obesity [60–65] (Figure 1). The mutations or alterations in the genes associated with obesity were linked to several clinical consequences such as the defective immune system, low blood pressure, cognitive deficiency, hypopigmentation, insulin resistance, and metabolic dysfunction [65].

**Figure 2:**
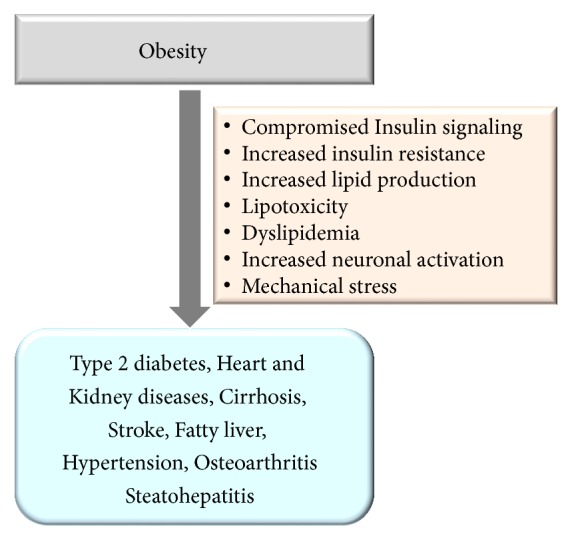
The major consequences of obesity.

Heymsfield and Wadden [65] have reviewed the pathophysiological consequences of obesity in detail. The lethal obese condition accelerates the incidence of type 2 diabetes (T2D) via increased adipokine, proinflammatory cytokines synthesis, and impaired insulin signaling and increased insulin resistance. The increased lipid production in obese condition releases free fatty acids, which cause lipotoxicity and chronic diseases like T2D [66] and other disease conditions such as cirrhosis, fatty liver, steatohepatitis, stroke, and heart failure [67]. Also, the accelerated sympathetic nervous system and renin-angiotensin-aldosterone system cause systemic hypertension, which ends up with several chronic and heart diseases [68]. Due to the overweight, internal organs of obese people become damaged by mechanical stress that causes overload on joints, and increased intra-abdominal tension leads to the development of osteoarthritis and gastroesophageal reflux disease [69, 70]. Obesity also leads to obstructive sleep apnea, which is due to the obstructions of the upper airway during sleep [71] (Figure 2).

## 3. Microbiome

GM represents densely populated microorganism such as bacteria, fungi, Archaea, protozoa, and viruses, which colonizes the human gastrointestinal tract. Approximately 100 trillion microbes colonize the human gut, which exhibits a symbiotic relationship with the host [72]. Each individual has unique GM composition influenced by several endogenous and exogenous factors such as gestational age, mode of delivery, breastfeeding, antibiotic exposure, diet, and lifestyle [73]. The colonization of GM is not uniform throughout the gastrointestinal tract with limited distribution in stomach and small intestine followed by a dense and diverse population in the colon due to the absence of digestive secretion, slow peristalsis, and rich nutrient supply [74]. These microbes play a significant role in maintaining host body homeostasis by participating in the digestive process and energy production, hampering pathogen colonization, and modulating the immune system.

Gut microbiome influences the individual's metabolic ability such as caloric extraction from indigestible dietary substance and its storage in adipose tissue, which predisposes an individual to obesity. Studies in germ-free and conventional mice showed alteration in kidney, liver, and intestinal homeostasis in germ-free mice depicting the fact that gut microbiome influences the whole body metabolism [75–77]. GM has received much attention related to human health and disease status in the recent decade. Conventionally, the interrelation between genetic and environmental factors such as high-calorie diet and lack of physical activity was considered as main contributor to obesity. However, recent scientific investigations have shown that GM has emerged as a prime endogenous factor influencing obesity [78, 79].

### 3.1. Development and Composition of Gut Microbiota

Emerging studies showed that prenatal gut microbiome represents the maternal microbes transmitted to the fetus through placental circulation and its composition acts as a determining factor for offspring microbial composition. Maternal obesity during pregnancy together with GM dysbiosis reflects in offspring microbiota leading to offspring metabolic disorders. GM of the newborn is also influenced by the factors like mode of birth, antibiotic treatment, feeding type, and sanitation [80]. In the first year of child life, the microbial composition varies according to developmental changes, host genotype, and food intake, which stabilizes similar to adult microbiota by the age of 3 years. The adult human gut is colonized by 10^14^ bacteria with billions of genes exceeding the human genome content. These microbial factories contribute to biochemical and metabolic function in the human body, which cannot be performed in its absence. In healthy adult individuals, the microbiota of the gut is in a symbiotic relationship with the host, which depends on host lifestyle, diet, and antibiotics, while in elderly people the composition of microbiota changes depending upon the alteration in digestive physiology and diet [81]. GM belongs to the phyla Bacteroidetes, Firmicutes, Actinobacteria, Proteobacteria, and Verrucomicrobia among which, Bacteroidetes and Firmicutes account for 90% of the total bacterial species [82]. Type and density of the bacterial population in gastrointestinal (GI) tract depend on environmental variation such as pH, oxygen level, and nutrient availability. Recent findings in animal and human models revealed that the GM plays a key role in nutrient acquirement, energy harvest, and host metabolic pathways, which are interrelated and are responsible for the development of obesity [83, 84]. Healthy human GM is characterized by a high ratio of Bacteroidetes to Firmicutes, while in obese individuals inverse ratio is observed with a high prevalence of Firmicutes [85]. In addition, the elevated level of* Lactobacillus* species with a relatively low level of* Bacteroides vulgatus* was observed in obese individuals [86]. Metagenomic analysis and clinical studies on GM of lean and obese individuals exhibited diminished proportion of Bacteroidetes and increased level of Actinobacteria with no significant difference in Firmicutes revealing the fact that ratio of Firmicutes to Bacteroidetes acts as a biomarker of obesity susceptibility [87, 88]. These data strongly link that certain bacterial phyla/class/species colonized in the gut acts as a driving force leading to the onset of obesity.

### 3.2. Host-Gut Microbiota Metabolite Interaction

Mounting evidence revealed that metabolites derived by fermentation of food by GM play a vital role in regulating the host metabolism with perspective to obesity.* Clostridium* and* Eubacterium *belonging to GM convert bile acid in the intestine to its secondary forms such as deoxycholic acid and lithocholic acid which binds to TGR5 receptor (G-protein-coupled receptor) and stimulates the secretion of incretin hormone GLP-1 and insulin, thereby promoting the energy expenditure [89]. Long chain fatty acid such as linoleic acid derived by the GM modulates the lipid profile leading to adiposity [90]. Another important by-product of gut microbial fermentation is short chain fatty acids (SCFs) formed by the gut microbial digestion of indigestible poly- and oligosaccharides that escape from the digestion and absorption in the proximal jejunum [91]. SCFs primarily acetate and propionate produced by Bacteroidetes and butyrate contributed by Firmicutes regulate the host metabolism by influencing energy harvest, fat accumulation, and appetite [92]. SCF in the GI tract reduces the luminal pH enhancing the nutrient absorption and also acts as a carbon source for GM [93]. Butyrate, the prime energy source for colonocytes, promotes proliferation and maturation of colonocytes maintaining colon healthy. In addition, butyrate protects the colon by enhancing the expression of mucin 2 and modulating immune response [94]. Acetate and propionate cross the epithelium to the liver, where the propionate gets metabolized, while acetate alone remains in the peripheral circulation [95]. SCF plays a significant role in maintaining the epithelial barrier integrity by regulating the tight junction protein (claudin-1, occludin, and Zonula Occludens-1), while downregulation of these proteins leads to translocation of bacteria and LPS triggering an inflammatory response [96]. So apart from the energy source, SCF modulates host biological response such as inflammation, oxidative stress, and immune response to fight against intestinal diseases such as Crohn's disease, ulcerative colitis, and colorectal cancer [97, 98].

SCFs influence the host metabolism either by direct activation of G-coupled receptors such as free fatty acid receptors 2 and 3 (FFAR2/GPR41 and FFAR3/GPR41) expressed primarily in the gut epithelial cells or by inhibiting nuclear class I histone deacetylases (HDACs) within the epithelial cells [97]. Acetate binds to FFAR2 while butyrate and propionate bind to FFAR3 receptor regulating the level of satiety hormones ghrelin (orexigenic peptide), peptide-1 (GLP-1), and peptide YY (PYY) (anorexigenic peptide) [99]. Ghrelin is secreted before a meal, while GLP-1 and PYY are released into circulation after meals, which stimulates insulin secretion by pancreatic *β* cells, reduces food intake, and normalizes weight loss and energy intake. Increased production of SCFs increases the gut peptide PYY and GLP-1 together with a decrease in ghrelin leading to increased satiety and reduced food intake [100]. Butyrate and propionate also reduce the appetite by

- inducing the expression of leptin in adipocytes and regulating body weight and energy homeostasis by reducing food intake and increasing energy expenditure [101]
- inducing the expression of intestinal gluconeogenesis gene promoting gluconeogenesis [102]
- inhibiting histone acetyltransferase and deacetylases exhibiting anti-inflammatory phenotype, epigenetically inducing the immune cell proliferation and differentiation, and upregulating adiponectin mediated AMPK pathway promoting mitochondrial biogenesis and fatty acid oxidation [103]

SCF derived from GM regulates host metabolism by interaction with complex metabolic pathways intertwined with the nervous, endocrine, and immune system. In healthy individuals SCF modulates the gut integrity, gut hormone production, and immune function, while in diseased state SCF exhibits a protective effect against diabetes, ulcerative colitis, colorectal cancer, and neurodegenerative disorders [94, 104]. Understanding the mechanism of interaction of SCFs with its receptor will help in exploring the therapeutic way for the treatment of obesity and health-related disorders.

### 3.3. Gut Microbiota and Obesity

The interrelation between GM and host obesity was first reported by Wostmann et al. [105] based on their studies in germ-free (GF) rodents, i.e., animals devoid of bacteria and conventional ones. However, the mechanism behind the report was elucidated by Jeffery Gordon and his colleagues [2] who observed an increase in total body and gonadal fat in conventional mice when compared to GF mice consuming more food. Colonization of GF mice with cecum-derived microbiota showed an increase in body fat mass together with insulin resistance, adipocyte hypertrophy, and enhanced level of circulating leptin and glucose level. The possible mechanism involved might be (1) degradation of indigestible polysaccharide by GM increasing hepatic lipogenesis in the host and (2) suppressing intestinal expression of angiopoietin-like 4 (ANGPTL4), the inhibitor of lipoprotein lipase (LPL) thereby blocking the fatty acid metabolism leading to increased cellular uptake of fatty acids and adipocyte triglycerides accumulation [2, 106, 107]. GF mice fed with high fat and sugar diet exhibited lean phenotype while conventional mice fed with the same diet were observed to be obese. GF mice showed enhanced sensitivity to insulin improving glucose tolerance and exhibited altered cholesterol metabolism reducing the storage and enhancing fecal excretion of cholesterol.

GM leads to host obesity through various routes such as by altering the intestinal permeability leading to endotoxemia, enhanced calorie provision, and endocannabinoid system (eCB) stimulation and by regulating the lipid metabolism by enhancing lipoprotein lipase activity and lipogenesis.

Experimental studies in animals and human volunteers revealed that increased production of SCFs by GM provides additional calories to host leading weight gain [108]. Binding of these SCFs to GPR induces the secretion of peptide hormone PYY, which reduces the intestinal transit time increasing the nutrient absorption in the intestinal lumen leading to weight gain [109]. Feces of obese individuals showed an increased level of SCF when compared to lean individuals. However, Ibrügger et al. [110] illustrated that consumption of food rich in dietary fibers increases the SCF production, thereby significantly reducing the weight in contradiction to the previous hypothesis, which makes the role of SCF in obesity a puzzle.

Microbiota influences the LPL activity by altering the expression of fasting-induced adipose factor (FIAF), the inhibitor of LPL activity causing accumulation of triglycerides (TG) in adipocytes [2]. Increased level of TG in adipose tissue causes hypertrophy leading to chronic inflammation, preventing further deposition of TG in adipose tissue and thereby promoting ectopic accumulation of TG in other organs developing insulin resistance [111].

**Figure 3:**
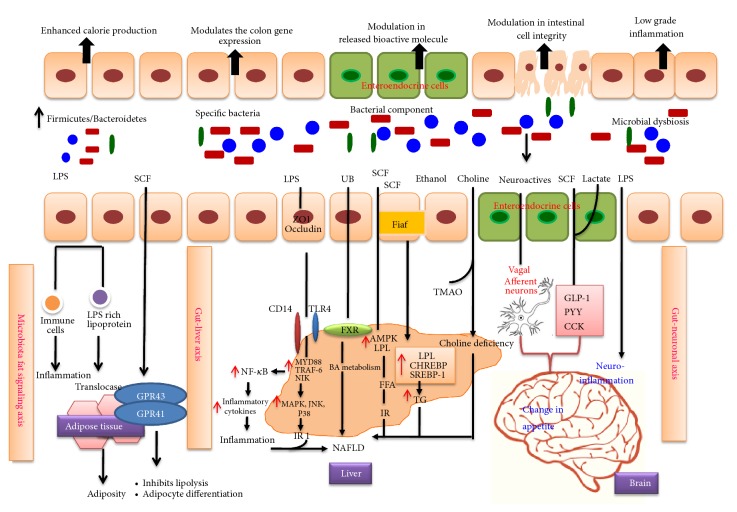
Gut microbial communication with immune cells and cells of target organs, pathways leading to obesity. Microbiota adipose tissue axis: metabolites of gut microbiota promote adipogenesis by triggering LPS based inflammation and SCF induced adipocyte differentiation. Gut-liver axis: microbiota dysbiosis alters the gut permeability enhancing the release of bacteria derived bioactive molecules in the liver. LPS interacts with TLR4 of the Kupffer cells enhancing the recruitment of MyD88 protein, IRAK, TRAF6, and NIK which promotes activation of MAPK, JNK, p38, and NF-*κ*B signaling pathways leading to inflammation and insulin resistance ultimately causing nonalcohol fatty liver (NAFLD). Metabolites like bile acid, SCF, and choline play a vital role in causing NAFLD. Gut-brain axis: neuroactive peptides, lactate, SCF, and LPS of gut microbiota activate vagal afferent neurons and gut hormones leading to alteration in appetite and neuroinflammation.

Lipopolysaccharides (LPS), the cell membrane component of Gram-negative bacteria, act as triggering factors leading to low-grade chronic inflammation followed by the development of insulin resistance (IR). LPS formed in the gastrointestinal tract reach the circulation via direct diffusion by enhancing the intestinal permeability or through absorption and incorporation with chylomicron [112]. Enhanced level of LPS in circulation is called endotoxemia where diet plays a key role. High fat intake inhibits the expression of tight junction proteins zonulin and occludin, thereby increasing intestinal permeability of LPS, the causative factor for endotoxemia. LPS interact with toll-like receptors TLR-4 in immune cells and target organs like liver and adipose tissue. LPS interaction with TLR-4 induces conformational change promoting the recruitment of adapter molecules like MyD88 protein, IRAK, TRAF6, and NIK to intracellular domain, thereby stimulating the phosphorylation and degradation of IKKB, the NF-*κ*B inhibitors. Translocation of active NF-*κ*B to the nucleus activates the expression of inflammatory proteins and also triggers signaling pathways like JNK, p38 MAPK, and ERK which induces insulin resistance leading to obesity (Figure 3). Administration of* Bifidobacterium infantis* in mice reduced colonic permeability attenuating inflammation revealing that gut microbial composition also plays a role together with diet in altering the intestinal permeability. Excess dietary lipid intake not only increases systemic exposure to potentially proinflammatory free fatty acids and their derivatives but more specifically facilitates the absorption of endotoxins, leading to higher plasma LPS level termed as “metabolic endotoxemia” [113, 114].

eCB modulates the food intake by regulating the expression of anorexigenic and orexigenic mediators such as endocannabinoids (endogenous lipids like N-arachidonyl ethanolamine (AEA) and glycerol 2-arachidonoyl (2-AG) and cannabinoid receptor (CB1 and CB2) coupled with G2 protein). AEA and 2-AG were synthesized using phospholipase D enzyme (NAPE-PLD) and sn-1-diacylglycerol lipase selective (DAG lipase) dependent on phospholipids and are metabolized into inactive compounds by fatty acid amide hydrolase (FAAH) and monoacylglycerol lipase (MGL). Interaction of endogenous lipid with cannabinoid receptor (CB1 and CB2) activates adenylate cyclase and also stimulates secondary messenger involved in MAPK, ERK, and NF-*κ*B pathway, promoting inflammation and insulin resistance, ultimately leading to obesity [115]. Experimental studies showed an increased concentration of AEA, NAPE-PLD, and CB1 and low expression of FAAH in the adipose tissue, while the reverse was observed in prebiotic treated animals revealing the fact that eCB activation leads to obesity and intervention of eCB upregulation is beneficial [114, 116]. Overall the studies reveal that GM activates the eCB system, which increases intestinal permeability promoting LPS migration into the circulatory system causing endotoxemia. Increased LPS, in turn, alters the tight junction integrity of the intestinal membrane enhancing increased release of LPS into circulation creating virtuous circle promoting adipogenesis.

Although research on human GM has succeeded logarithmically, still this field remains a puzzle and is emerging which needs to be explored. The gut microbiome is a complex microbial world having both beneficial and harmful microbes and manipulation of these microbes for the therapeutic purpose is possible only if the precise role of each and every individual microbe is known. GM, its metabolite, and host are interplaying systems; therefore integration of this system will give us a comprehensive idea of the function of each building block of this system [117, 118].

## 4. Influence of Probiotic Supplementation on Health Status of Obese People

Intervention of calorie restricted diet (1500 kcal per day) supplemented with cheese (50 g per day) containing probiotic (*Lactobacillus plantarum* TENSIA; 8.7 log CFU per g) for three weeks significantly reduced the body mass index (BMI) in patients with obese and hypertension compared to the control group (patients fed calorie restricted diet supplemented with control cheese). The reduced morning systolic blood pressure was also observed in the patients of both groups treated with calorie restricted diet along with the aid of antihypertension drugs irrespective of the cheese (probiotic cheese or control cheese) consumption. The urinary putrescine content and BMI changes were associated with the lactobacilli load in the intervention group. The study suggested that supplementation of probiotic cheese with a calorie restriction diet reduces the BMI and hypertension in study subjects [24].

Overweight or obese adults were supplemented with* L. gasseri* BNR17 (10^10^ CFU per capsule; 6 capsules per day) (a probiotic strain isolated from human breast milk) for 12 weeks and the changes in body mass, body fat, behavior, and biochemical parameters were assessed at four different intervals (0, 4, 8, and 12^th^ week of intervention). The results suggested that the supplementation of BNR17 reduced body weight, hip, and waist circumferences compared to the placebo group. Other tested parameters such as gastrointestinal, genital, endocrine, respiratory, and diabetic associated parameters were not found to be changed during the study among the study subjects. The supplementation of BNR17 has not influenced the behavior pattern of the subjects, and no adverse effects were observed. The study suggested that the intervention of single strain probiotic reduced the body weight in obese people, and further in-depth extended research is necessary to explain the health benefits of the strain [25]. A recent study revealed that the supplementation of BNR17 (10^10^ CFU per day) significantly reduced the visceral adipose tissue and waist circumferences in obese adults [26].

Visceral adiposity of the healthy volunteers (with the large visceral fat area) has been significantly reduced after 12-week supplementation of fermented milk containing* L. gasseri* SBT2055 (200 g per day; 10^6^ or 10^7^ or 10^8^ CFU per g of milk) compared to control group. Also, the notable reduction was observed in body weight, BMI, and waist and hip circumferences of people who had the probiotic intervention [27, 28]. The intervention associated positive changes were diminished after 4 weeks of cessation of probiotic supplementation, which indicates that continuous ingestion of* L. gasseri* SBT2055, even at the low dose (10^6^ CFU per g), is necessary to reduce the obesity-associated consequences [28].

The healthy overweight people were randomly divided into different groups and were supplemented with VSL#3 (a probiotic formulation containing three strains of* Bifidobacterium* and four strains of* Lactobacillus*; 112.5 × 10^9^ CFU per capsule; one capsule per day) or omega 3 fatty acids (180 mg EPA and 120 mg DHA per day) or both VSL#3 and omega 3 fatty acids for 6 weeks. After 6 weeks of supplementation, total cholesterol, low-density lipids (LDL), very-low-density lipids (VLDL), TG, and high-sensitivity C-reactive protein (hsCRP) were observed to be significantly reduced in VSL#3 supplemented group. Additionally, VSL#3 supplementation improved the high-density lipids (HDL) level and insulin sensitivity. The positive regulation of GM was also observed in probiotic-supplemented groups. Omega 3 fatty acids supplementation also showed improved insulin sensitivity, a slight reduction in LDL and hsCRP levels, and no effect on the composition of the GM. The combination of VSL#3 and omega 3 fatty acids showed more pronounced effects. The high hsCRP levels and low HDL level were correlated with the high concentration of Bacteroides and low* Bifidobacterium* and* Lactobacillus* content in study subjects [29].

The obese pregnant women were supplemented with a single probiotic strain of* L. salivarius* UCC118 (10^9^ CFU per capsule; one capsule per day) for four weeks from the 24^th^ week of gestation. The results suggested that UCC118 supplementation reduced the BMI of obese pregnant women compared to placebo control, but no changes were observed in impaired glycemia incidences, metabolic profile, and pregnancy outcomes [30]. In another study, the obese pregnant women were supplemented with Vivomixx® (a mixture of* Bifidobacterium longum* DSM 24736,* B. breve* DSM 24732,* B. infantis *DSM 24737,* Streptococcus thermophilus* DSM 24731,* L. delbrueckii *subsp.* bulgaricus* DSM 24734,* L. acidophilus *DSM 24735,* L. paracasei* DSM 24733, and* L. plantarum* DSM 24730; 4.5 × 10^10^ CFU in total) from 14-20 weeks of gestation until the delivery of the baby. After the detailed analysis of blood, urine, fecal samples, and diet profile and weight gain, it has been proved that the supplementation of Vivomixx® significantly reduced the weight gain during the pregnancy period and reduced the pregnancy complications via positive alteration of GM of obese pregnant women [31].

The supplementation of* L. paracasei* F19 (9.4 × 10^10^ CFU per day) for six weeks had no effect on the GM and metabolic profile of obese postmenopausal women. However, the intervention of flaxseed mucilage (10 g per day) for six weeks reduced the serum C-peptide, increased the insulin sensitivity, and altered the abundance of about 33 metagenomic species in obese postmenopausal women. The improved insulin sensitivity is not associated with altered microbiome [32]. Likewise, the supplementation of low (2.5 × 10^9^ CFU per day) and high dose (1× 10^10^ CFU per day) of multistrain probiotic preparation Ecologic® (*B. bifidum* W23,* L. salivarius* W24,* L. acidophilus* W37,* B. lactis* W51,* B. lactis* W52,* L. casei* W56,* L. brevis *W63,* Lactococcus lactis* W19, and* L. lactis* W58) for 12 weeks showed a health improvement in obese postmenopausal women. Intake of a high dose of probiotic reduced the lipopolysaccharide, fat mass, glucose, HOMA-IR index, LDL, subcutaneous fat, total cholesterol, TG, insulin, uric acid, and waist circumference in the studied subjects. The results claimed that supplementation of multistrain probiotic preparations improved the cardiometabolic parameters and intestinal permeability in obese postmenopausal women [33].

The overweight adults were supplemented with* B. breve *B-3 (5 × 10^10^ CFU per day) for 12 weeks and the metabolic parameters and adiposity level were measured. The results indicated that B-3 supplementation reduced the fat mass in subjects and also improved the blood parameters associated with liver function and inflammatory system in the studied overweight adults [34].

The influence of VSL#3 on a high-fat diet (HFD) induced obesity has been assessed. The healthy nonobese adults were supplemented with HFD (diet containing 55, 30, and 15% of fat, carbohydrate, and protein, respectively, and it provides extra ~1000 kcal per day) and VSL#3 (4.5 × 10^10^ CFU per day) for four weeks and the changes in body mass and fat content were measured. The results revealed that the supplementation of VSL#3 significantly prevents the development of excess body and fat mass in the subjects compared to the placebo control group [35].

The supplementation of* B. animalis* ssp.* lactis* 420 (B420) (10^10^ CFU per day) with or without fiber (Litesse® Ultra polydextrose; 12 g per day) for six months significantly reduced the fat mass in overweight and obese adults. The fat mass in the abdominal region and waist circumference was reduced predominantly after B420 supplementation. The intervention of fiber alone showed no positive changes in the subjects. The reduction of blood hsCRP and zonulin level was related to the changes in trunk fat mass. The study results revealed that supplementation of B420 alone was enough to reduce the fat mass in studied subjects [36].

The influence of supplementation of probiotic yogurt (PY) and regular low-fat yogurt (LFY) on weight loss program has been studied in obese and overweight women. The consumption of PY (containing* B. lactis* BB12 and* L. acidophilus* LA5; 10^7^ CFU per day) for 12 weeks significantly reduced the total cholesterol, LDL, and insulin resistance whereas no notable changes were observed in body mass, HDL, fasting plasma glucose, and TG level. The results suggested that the consumption of PY along with regular diet had not greatly influenced weight reduction, but it improves the lipid profile and insulin sensitivity in the obese and overweight women [37].

The supplementation of probiotic mix (*L. rhamnosus* DSMZ 21690 (2 × 10^9^ CFU),* L. acidophilus* ATCCB3208 (3 × 10^9^ CFU),* B. bifidum* ATCC SD6576 (2 × 10^9^ CFU), and* B. lactis* DSMZ 32269 (6 × 10^9^ CFU) per day) for 12 weeks improved the liver profile in obese children and adults with nonalcoholic fatty liver disease. After the probiotic supplementation, the level of alanine aminotransferase and aspartate aminotransferase was decreased significantly in the probiotic group. The cholesterol, LDL, TG, and waist circumference were also reduced with notable level while body weight, BMI, and fat mass were not changed. And based on the sonography results, the study suggested that the supplementation of probiotic improved the liver conditions in the subjects [38].

The supplementation of the probiotic mix (Danisco®;* B. lactis*,* B. bifidum*,* L. casei*,* L. acidophilus*, and* Lactococcus lactis*; 2 × 10^10^ CFU per day) and/or controlled diet for 8 weeks significantly reduced the polyunsaturated fatty acids level, conicity index, waist-height ratio, and waist circumference and increased the glutathione peroxidase activity in obese or overweight women. The results suggested that the supplementation of probiotic mix and controlled diet improved the antioxidant system of the subjects and effectively reduced the obesity-associated consequences compared to that of the intervention of controlled diet (without a probiotic mix) in the placebo group [39].

The overweight people were supplemented with probiotic preparation containing* L. plantarum* KY1032 and* L. curvatus* HY7601 at the concentration of each 2.5 × 10^9^ CFU per day for twelve weeks. The level of dodecenoylcarnitine, decanoylcarnitine, tetradecenoylcarnitine, and octanoylcarnitine was found to be increased, while the body weight and fat mass were reduced in the probiotic-supplemented group. The study claimed that the positive effect of probiotic intervention was attributed to the increase in medium-chain acylcarnitines in the studied overweight individuals [40].

The supplementation of a mixture of probiotic strains (*B. bifidum*,* B. longum*,* B. infantis*,* L. acidophilus*,* L. casei*, and* L. lactis*; 3 ×10^10^ CFU per day) for four weeks had no significant improvement in the waist circumference, body mass, blood glucose level, and fecal short-chain fatty acid in the studied overweight people. But the reduction in energy intake was clearly noted in the probiotic-supplemented group compared to baseline value. The study suggested that probiotic preparation can be used in diet management for weight loss program as an adjuvant [41].

A twelve-week supplementation of synbiotic preparation (*L. rhamnosus *CGMCC1.3724 (3.24 ×10^8^ CFU, 90 g inulin, and 210 g oligofructose per day)) effectively induced weight loss in obese women. The disinhibition and hunger scores, Beck Depression Inventory score, and food craving were reduced, while the satiety efficiency and Body Esteem Scale were increased in the studied obese women. The male subjects also displayed positive effects like fasting fullness and cognitive restraint. The study suggested that the supplementation of synbiotics controls appetite and associated behavior in obese people during weight management [42].

The probiotic oral suspension (psychobiotics) was supplemented to normal weight lean (BMI <25 kg/m^2^ and total body fat % < 30), normal weight obese (BMI <25 kg/m^2^ and total body fat % ≥ 30), and preobese-obese (BMI ≥ 25 kg/m^2^ and total body fat % ≥ 30) women for three weeks. The probiotic formula consists of 1.5 ×10^10^ CFU per strain of* B. bifidum* SGB02,* B. animalis *subsp.* lactis* SGB06,* S. thermophilus* SGSt01,* S. thermophiles*,* L. plantarum *SGL07,* L. delbrueckii *spp.* bulgaricus *DSM 20081,* L. reuteri* SGL01,* L. acidophilus *SGL11, and* Lactococcus lactis *subsp.* lactis *SGLc01. The supplementation significantly reduced the BMI, total fat mass, psychopathological scores, bacterial overgrowth syndrome, and body uneasiness test and global severity index scale while it improved the free fat mass, meteorism, and defecation frequency in preobese and normal weight obese subjects compared to baseline [43].

The morbid obese patients who underwent Anastomosis Gastric Bypass-Mini Gastric Bypass (OAGB-MGB) surgery were supplemented with probiotic preparation, starting from 4 weeks before the surgery to 12 weeks after surgery. The probiotic formula Familact® contains* L. casei* (3.5 × 10^9^ CFU),* L. rhamnosus* (7.5 × 10^8^ CFU),* L. bulgaricus* (10^8^ CFU),* L. acidophilus* (10^9^ CFU),* B. breve* (10^10^ CFU),* B. longum* (3.5 × 10^9^ CFU), and* S. thermophilus *(10^8^ CFU) and fructooligosaccharide (38.5 mg) in one serving sachet. After 16 weeks of supplementation of one sachet per day, the weight loss efficiency, anthropometric quantities, vitamin D status, and inflammatory system significantly improved in OAGB-MGB patients without affecting the folate, vitamin B_12_, and homocysteine levels. Further studies are required to confirm the efficiency of the probiotic supplementation in detail [44].

Healthy preobese people (BMI ≥ 25 kg/m^2^ but BMI < 30 kg/m^2^) were supplemented with* B. breve* B-3 (2 × 10^10^ CFU per day) for 12 weeks, and the changes in the baby fat mass, body weight, and blood parameters were measured.* B. breve* B-3 supplementation effectively reduced the body fat mass and TG and improved the HDL level in preobese people compared to baseline. The study suggested that the regular supplementation of* B. breve* B-3 helps to reduce the body fat mass [45].

The supplementation of fortified yogurt (prepared with* S. thermophiles *and* L. bulgaricus *as starter culture and enriched with 10^7^ CFU of* B. lactis Bb-12* per gram, inulin, whey protein, vitamin D_3_, and calcium) to obese individuals significantly improved the body composition (reduced the waist circumference, body fat percentage, body fat, TG level, and Homoeostasis Model of Assessment-Insulin Resistance (HOMA-IR) value and increased the HDL, 25-hydroxyvitamin D level, and Quantitative Insulin Sensitivity Check Index (QUICKI)) and metabolic profile. The level of free fat mass was not reduced significantly in the fortified yogurt supplemented group compared to the low-fat plain yogurt (prepared with* S. thermophiles *and* L. bulgaricus *as starter culture) group. The positive effects of fortified yogurt were associated with calorie restricted diet plan during the intervention period. The study suggested that regular consumption of probiotic-enriched fortified yogurt along with a strict diet plan helps to reduce the body weight and to improve the metabolic status in obese people [46].

The influence of supplementation of live and heat-killed* B. animalis* subsp.* lactis* CECT 8145 on the health status of obese people has been reported by Valls group. The study revealed that the consumption of 10^10^ CFU of live cells of the studied probiotic per day significantly reduced the BMI, visceral fat, conicity index, waist circumference, and waist circumference/height ratio and increased the population of* Akkermansia* spp. in the GM of the obese adults. The consumption of heat-killed cells reduced the blood pressure and HOMA index. The predominant probiotics effects were observed in women subjects, and changes were significant compared to baseline value and placebo control. Further studies are needed to reveal the correlation between change in* Akkermansia* spp. abundance in GM and altered body composition during the CECT 8145 supplementation in obese subjects [47].

The supplementation of* B. pseudocatenulatum *CECT 7765 (10^9-10^ CFU per day for 13 weeks) significantly reduced the BMI, hsCRP, and monocyte chemoattractant protein-1 and increased the omentin-1 and HDL level in insulin-resistant obese children. The microbiome analysis revealed that the supplementation of CECT 7765 increased the* Alistipes* spp. in GM of the studied subjects. The study claimed that the positive effects of CECT 7765 were attributed to the increase in* Rikenellaceae* family members, known to associate with the lean phenotype [48] (Table 1).

## 5. *In Vivo* Studies Using Laboratory Animal Models

The influence of supplementation of probiotic strain* L. rhamnosus* PB01 (DSM 14870) on sperm kinetics in diet-induced obese mice has been reported by Dardmeh group. The study suggested that the supplementation of DSM 14870 (1 × 10^9^ CFU per day for 4 weeks) effectively improved the levels of serum testosterone, follicle-stimulating hormone, and luteinizing hormone. The concentration of highly active motile sperm was increased and a significant level of reduction in nonmotile sperm count was observed in both obese and lean (normal weight) mice models. The sperm kinetic measurements revealed that DSM 14870 intervention remarkably increased the sperm kinetics in obese mice model compared to baseline values. The study suggested that the supplementation of DSM 14870 improved the sperm motility, reproductive hormones levels, and weight loss in diet-induced obese mice model [49].

The combination of probiotic supplementation (*L. rhamnosus*; 1 × 10^8^ CFU per day) and ultrasound treatment (at acoustic pressure of 2 W/cm^2^ for 30 sec) for 8 weeks significantly reduced the body mass, total body fat mass, and thickness of the subcutaneous fat layer and improved the plasma lipid profile in diet-induced obese mice model compared to baseline and single treatment groups (either probiotic supplementation or ultrasound treatment) [50].

The supplementation of* L. reuteri *263 (2.1 × 10^9^ or 1.05 ×10^10^ CFU per day for 8 weeks) altered the energy metabolism in white adipose tissue of high-energy-diet-fed rat model. The expression of genes associated with thermogenesis, glucose, and lipid metabolism has been altered significantly after the intervention of* L. reuteri *263. The level of proinflammatory markers and antioxidant system of the host has been modified and confers the protection against the high-energy-diet-induced consequences. The study revealed that the antiobesity activity of* L. reuteri *263 was attributed to the ability of remodeling of energy metabolism in white adipose tissue of high-energy-diet-fed rat model [51].

The supplementation of* Lactobacillus *strain (*L. plantarum* and/or* L. fermentum*; 1 × 10^8^ CFU per day for 8 weeks) improved the systemic immune status of the HFD fed rat model. Fat vehicle sizes, liver steatosis, endotoxin, and IL-6 level were significantly reduced in the probiotic-supplemented group compared to control. The microbiota analysis revealed that the supplementation of probiotics notably improved* Lactobacillus* and* Bifidobacterium* content in an experimental model. The study claimed that the combination of multiple strains of probiotics confers better health benefits in obese experimental models via modulating the intestinal microbiota and immune system when compared to single strain intervention [52]. The supplementation of soy-based probiotic product (containing* B. longum* ATCC 15707 and* Enterococcus faecium* CRL 183) altered the intestinal microbiota and immune profile in a positive way in HFD-induced obese (HFDO) mice model [53]. Similarly, the supplementation of a multistrain probiotic preparation (*L. rhamnosus* LR5,* L. acidophilus* LA1,* S. thermophilus* ST3,* B. longum* BG7,* B. lactis* BL3, and* B. bifidum *BF3) for 8 weeks significantly reduced the body weight and improved the serum level metabolic profile in the HFDO rat. The microbiota analysis showed that probiotic intervention increased the amount of* Lactobacillus*,* Bacteroidetes, *and* Bifidobacterium *while it reduced the* Firmicutes *load in experimental animals [54] (Table 2).

## 6. Conclusion and Future Perspectives

The detailed literature survey showed that the beneficial impact of probiotic supplementation in obese people has been associated with several factors such as nature of the probiotic strain, composition of the probiotic formula (single or multistrain; with or without prebiotics), duration of the intervention, dose, and other aided activities like calorie/dietary restrictions and weight loss medications.

A meta-analysis study reported that the probiotic supplementation was not associated with weight loss in obese people, which may be due to the less number of articles (studies with clinical trials) chosen for the analysis based on the selection criteria (randomized controlled trials; supplementation of probiotic; control (placebo or no probiotic supplementation); results of body weight and BMI) of the study [119]. Nevertheless, the subsequent report based on a meta-analysis of 25 clinical trials with 1931 obese subjects revealed that the probiotic supplementation effectively reduced the body weight. The study also disclosed that a minimum of 8 weeks of multistrain probiotic intervention reduced the body weight in obese subjects compared to single strain intervention and fewer intervention periods [120]. A recent systemic review and meta-analysis of fifteen clinical trial studies with 957 subjects revealed that the intervention of probiotic supplementation for 3 to 12 weeks significantly reduced the body weight and fat mass in obese subjects compared to placebo group [121].

**Figure 4:**
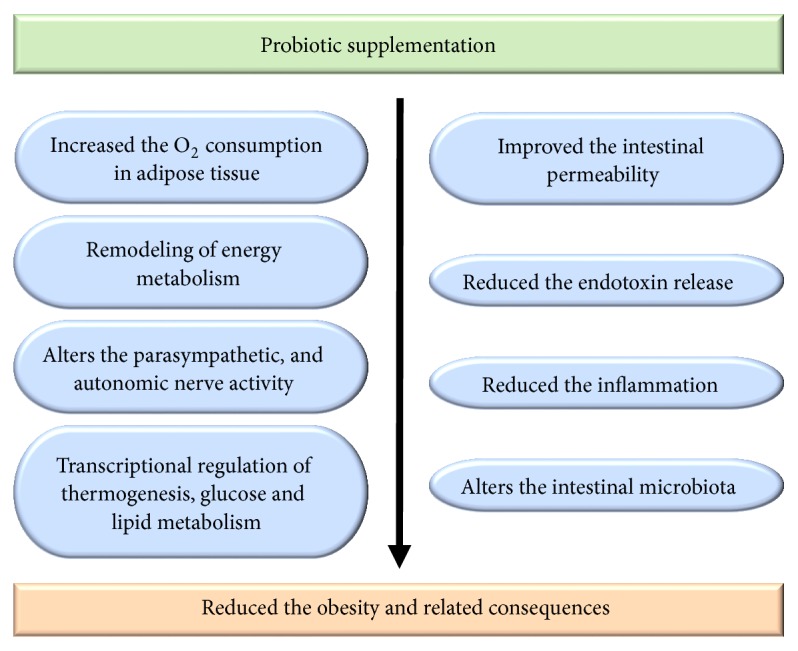
The possible mechanism behind the antiobesity property of probiotics.

The antiobese activity of probiotic supplementation may be associated with the ability to remodel the energy metabolism, alter the expression of genes related to thermogenesis, glucose, and lipid metabolism genes, enhance the intestinal permeability, reduce the release of endotoxins, reduce the inflammation, and change the parasympathetic nerve activity. Most importantly probiotic intervention greatly modified the composition of intestinal microbiota (increased the load of* Bifidobacterium*,* Lactobacillus*, Proteobacteria, Bacteroidetes, and Peptococcaceae members and reduced the amount of Firmicutes, Clostridium, and Actinobacteria), which accelerates the weight loss in obese people [122] (Figure 4).

Interrelationship between the dietary supplement and GM envisages the fact that “we are what we eat and what our gut microbiome is.” In the near future, several gut-microbiome based clinic will become prevalent where the individual's GM will be used for diagnosis, prophylaxis, and therapy of human health problems. In addition, analysis of the infant's gut microbial composition will help us to predispose the future ailments based on which the individual diet can be designed for better and disease-free health and well-being. To conclude, as GM plays a key role in host metabolism, modulation of its composition represents a promising strategy for the treatment of obesity. The results of recent studies revealed that the supplementation of probiotic formulations improved the health status of obese people, and the regular consumption of probiotic is advisable to retain the health benefits. Further studies are necessary to evaluate the best combination of probiotic strains or synbiotic preparation to extend the health benefits of probiotics in obese individuals.

## Figures and Tables

**Table 1:** Influence of probiotic supplementation on obese people.

Study subjects	Intervention	Dose	Duration of the study	Clinical outcomes	Conclusions	Ref.
People with obesity and hypertension	Hypocaloric diet (1500 kcal/d), and cheese containing *Lactobacillus plantarum* TENSIA	Calorie restricted diet and 50 g of cheese per day	3 weeks	↓BMI BMI associated with lactobacilli load in the intestine. ↓ Morning systolic BP.	Calorie restriction and probiotic supplementation significantly reduced the BMI, and systolic BP in people with obesity and hypertension.	[24]

Overweight or obese people	*L. gasseri* BNR17	6 capsules per day (10^10^ CFU per capsule)	12 weeks	↓ Weight, waist and hip circumferences.	Reduced the body weight, waist and hip circumferences compared to baseline.	[25]

Obese people	*L. gasseri* BNR17	10^10^ CFU per day	12 weeks	↓ Visceral adipose tissue, waist circumferences.	The probiotic supplementation reduced the visceral fat accumulation and waist circumference.	[26]

Adults with abdominal adiposity	*L. gasseri* SBT2055 mediated fermented milk (FM)	200 g of FM per day (10^6^ / 10^7^ / 10^8^CFU per g of FM)	12 weeks	↓ Abdominal visceral fat ↓ Body weight, waist and hip circumferences ↓BMI	Low concentration of SBT2055 supplementation reduced the obesity associated health problems, but continuous consumption is needed to maintain the effect.	[27, 28]

Healthy Overweight volunteers	VSL#3 and /or omega 3 fatty acid (OFA)	1 capsule per day (112.5 × 10^9^ CFU per capsule); 1 OFA capsule per day (180 mg EPA, and 120 mg DHA)	6 weeks	VSL#3 alone or in combination of OFA supplementation: ↓ Total cholesterol, triglyceride, LDL, VLDL, and hsCRP ↑ HDL level, insulin sensitivity. Altered the gut microbiota positively. OFA supplementation did not affect the microbiota.	The combination of VSL#3 and OFA showed more pronounced effects.	[29]

Obese pregnant women	*L. salivarius* UCC118	1 capsule per day (10^9^ CFU per capsule)	4 weeks	↓BMI No changes in glycemia, and pregnancy outcomes	UCC118 supplementation had not significantly affected the metabolic profile, fasting glucose level, and pregnancy outcomes.	[30]

Obese pregnant women	Vivomixx®	A mixture of probiotic strains (4.5 × 10^10^ CFU per day)	Depends*∗*	↓ weight gain and pregnancy consequences	Altered the microbiota in a positive way and reduced the complications of pregnancy and weight gain.	[31]

Obese postmenopausal women	*L. paracasei* F19 or flaxseed mucilage (FM)	9.4 × 10^10^ CFU per day or 10 g of FM	6 weeks	FM intervention altered the microbiota and improves insulin sensitivity. But F19 supplementation had no effect on the metabolic profile in the subjects	FM improved the health status of obese postmenopausal women	[32]

Obese postmenopausal women	Ecologic® (*Bifidobacterium bifidum* W23, *L. salivarius* W24, *L. acidophilus* W37, *B. lactis* W51, *B. lactis* W52, *L. casei* W56, *L. brevis *W63, *Lactococcus lactis* W19, and *L. lactis* W58)	High dose (1× 10^10^ CFU per day); Low dose (2.5 × 10^9^ CFU per day)	12 weeks	Level of LPS, fat mass, glucose, HOMA-IR index, LDL, subcutaneous fat, total cholesterol, TG, insulin, uric acid, waist circumference	Both high and low dose of Ecologic® supplementation improved the cardiometabolic parameters and intestinal permeability in obese postmenopausal women.	[33]

Overweight adults	*B. breve* B-3	5 × 10^10^ CFU per day	12 weeks	↓ Fat mass Improved the liver function, and inflammation system.	B-3 supplementation improved the metabolic profile of overweight adults	[34]

Healthy non-obese people	VSL#3 and high fat diet	4.5 × 10^10^ CFU per day	4 weeks	Reduced the body mass and fat mass compared to placebo control	VSL#3 supplementation prevents the high-fat diet and induced fat and body mass increase in healthy non-obese adults	[35]

Overweight and obese people	*B. animalis* ssp. *lactis* 420 (B420) with/ without Litesse® Ultra polydextrose (LU)	10^10^ CFU per day; 12 g of LU per day	6 months	↓ Fat mass, waist circumference. ↓ Blood hsCRP, and zonulin level	B420 supplementation reduced the fat mass in obese people with or without LU.	[36]

Obese and overweight women	Probiotic yogurt	A mixture of probiotic strains (10^7^ CFU per day)	12 weeks	No significant change in body mass, HDL, TG. ↓ Total cholesterol, LDL, insulin resistance.	Improved the insulin sensitivity, and lipid profiles in the obese and overweight women	[37]

Obese children and adults	Probiotic mix	*L. rhamnosus* DSMZ 21690 (2 × 10^9^ CFU), *L. acidophilus* ATCCB3208 (3 × 10^9^ CFU), *B. bifidum* ATCC SD6576 (2 × 10^9^ CFU), *B. lactis* DSMZ 32269 (6 × 10^9^ CFU) per day	12 weeks	↓ AsAT, AAT, cholesterol, LDL, TG, waist circumference	The probiotic supplementation improved the condition of nonalcoholic fatty liver disease in obese people	[38]

Obese or overweight women	Probiotic mix (Danisco®) and controlled diet	*B. lactis. B. bifidum, L. casei, L. acidophilus, Lactococcus lactis, *2 × 10^10^ CFU per day	8 weeks	↓ Polyunsaturated fatty acids level conicity index, waist-height ratio, waist circumference. ↑ glutathione peroxidase activity	Dietary restriction along with probiotic intervention effectively reduced the obesity associated consequences and improved the antioxidant status of the subjects	[39]

Overweight people	*L. plantarum *KY1032, and *L. curvatus* HY7601	2 g of probiotic powder (Each 2.5 ×10^9^ CFU per day)	12 weeks	↑ Dodecenoylcarnitine, decanoylcarnitine, tetradecenoylcarnitine, and octanoylcarnitine level. ↓body weight, body fat mass	Probiotic supplementation mediated weight loss is associated with increased medium-chain acylcarnitines in the overweight individuals.	[40]

Overweight people	Probiotic preparation (*B. bifidum, B. longum, B. infantis, L. acidophilus, L. casei, L. lactis*)	3 ×10^10^ CFU per day	4 weeks	No changes in waist circumference, body weight, glucose level. ↓ Energy intake	Probiotic supplementation reduced the energy intake compared to baseline. It can be used as an adjuvant for the weight loss program.	[41]

Obese people	Synbiotic formula (*L. rhamnosus* CGMCC1.3724, inulin, oligofructose)	3.24 ×10^8^ CFU per day; 90 g inulin, 210 g oligofructose per day	12 weeks	↑ Weight loss in women. ↑ Satiety efficiency↓ Disinhibition and hunger scores, food craving.	The intervention of synbiotic preparation influenced the appetite control and associated behavior in obese people during weight loss program	[42]

Preobese-obese, normal weight obese, and normal weight lean women	Psychobiotics (*B. bifidum* SGB02, *B. animalis *subsp. *lactis* SGB06, *Streptococcus thermophilus* SGSt01, *S. thermophiles*, *L. plantarum *SGL07, *L. delbrueckii *spp. *bulgaricus *DSM 20081, *L. reuteri* SGL01, *L. acidophilus *SGL11, *Lactococcus lactis *subsp. l*actis *SGLc01)	Each 1.5 ×10^10^ CFU per day	3 weeks	↓ BMI, Fat mass ↓ Psychopathological scores ↓ Bacterial overgrowth syndrome ↓ BUT_GSI scale ↑ Free fat mass Improved the meteorism and defecation frequency	Psychobiotics supplementation improved the body composition, reduced the dysbiosis, and reduced the psychopathological scores in preobese-obese and normal weight obese people.	[43]

Obese patients (OAGB-MGB)	Familact® *L. casei* (3.5 × 10^9^ CFU), *L. rhamnosus* (7.5 × 10^8^ CFU), *L. bulgaricus* (10^8^ CFU), *L. acidophilus* (10^9^ CFU), *B. breve* (10^10^ CFU), *B. longum* (3.5 × 10^9^ CFU), and *S. thermophilus *(10^8^ CFU), and fructo-oligosaccharide (38.5 mg) in one sachet	One sachet per day	4 weeks before surgery and 12 weeks after surgery	Improved the vitamin D status, inflammatory markers, lipid profile, and glycemic indices. No changes in folate, vitamin B_12_, and homocysteine levels	Familact® supplementation improved inflammatory markers and promoted weight loss in OAGB-MGB patients.	[44]

Healthy pre-obese adults	*B. breve* B-3	2 ×10^10^ CFU per day	12 weeks	↓ Body fat mass, TG ↑ HDL	B-3 supplementation reduced the body fat effectively in pre-obese subjects.	[45]

Obese people	Calorie restricted diet and fortified yogurt (*S. thermophiles, L. bulgaricus* and *B. lactis Bb-12*, inulin, whey protein, vitamin D_3_, calcium)	500 g of fortified yogurt per day	10 weeks	↓ Body fat mass ↓ Waist circumference, body fat percentage, TG level, HOMA-IR value. ↑ HDL, 25-hydroxyvitamin D level, QUICKI.	Improved the body composition, and metabolic profile in obese people	[46]

Abdominally obese people	*B. animalis* subsp. *lactis* CECT 8145	10^10^ CFU per day	12 weeks	↓ BMI, visceral fat ↓ Conicity index ↓ Waist circumference ↓ Waist circumference/ height ratio ↑ *Akkermansia* spp. in the gut microbiota	CECT 8145 supplementation effectively reduced the obesity associated consequences in abdominally obese people	[47]

Insulin-resistant obese children	*B. pseudocatenulatum *CECT 7765	10^9-10^ CFU per day	13 weeks	↓ BMI, hsCRP, monocyte chemoattractant protein-1 ↑ Omentin-1, and HDL. ↑ *Alistipes* spp.	CECT 7765 supplementation improved the lipid profile and inflammatory markers in obese children	[48]

*∗*Intervention starts from 14-20 weeks of the gestation and continued until delivery of the baby; ↓: Reduced; BMI: Body mass index; BP: Blood pressure; VSL#3: A probiotic formulation containing three strains of *Bifidobacterium* and four strains of *Lactobacillus*; EPA: Eicosapentaenoic acid; DHA: Docosahexaenoic acid; TG: Triglyceride; LDL: Low-density lipids; VLDL: Very-low-density lipids; HDL: High-density lipids; hsCRP: High-sensitivity C-reactive protein; Vivomixx®: A probiotic formulation containing eight bacterial strains; AAT: Alanine aminotransferase; AsAT: Aspartate aminotransferase; BUT_GSI scale: Body uneasiness test and global severity index; OAGB-MGB: Anastomosis Gastric Bypass-Mini Gastric Bypass; QUICKI: Quantitative Insulin Sensitivity Check Index; HOMA-IR: Homoeostasis Model of Assessment-Insulin Resistance; LPS: Lipopolysaccharide.

**Table 2:** Effects of probiotic supplementation on obese-experimental animal model.

Model system	Intervention	Dose and duration	Results	Conclusions	Ref.

Diet-induced obesity mice model	*Lactobacillus rhamnosus* PB01 (DSM 14870)	1 × 10^9^ CFU per day for 4 weeks	↑ Serum testosterone, FSH and LH levels. ↑ Sperm kinetics ↑ Highly active motile sperm level. ↓ Non-motile sperm count.	DSM 14870 supplementation increased sperm kinetics and amount of active motile sperm in diet-induced obese mice.	[49]

Diet-induced obesity mice model	*L. rhamnosus* and ultrasound	1 × 10^8^ CFU per day for 8 weeks	↓ Body weight, total body fat. ↓ Thickness of the subcutaneous fat layer. Improved the plasma lipid profile.	*L. rhamnosus* supplementation with ultrasound treatment effectively reduced the fat content and improved lipid profile in diet-induced obese mice.	[50]

High-energy-diet-fed rats	*L. reuteri *263	2.1 × 10^9^ CFU per kg per day for 8 weeks or 1.05 ×10^10^ CFU per kg per day for 8 weeks	↑ Proinflammatory factors and antioxidant enzymes. ↑ Oxygen consumption. ↑ Expression of genes associated with thermogenesis, glucose and lipid metabolism.	*L. reuteri *263 supplementation improved the obese condition via remodeling of energy metabolism in the high-energy-diet fed rat model.	[51]

High-fat diet rat	*L. plantarum* and *L. fermentum*	1 × 10^8^ CFU per day for 8 weeks	↓ Fat vehicle sizes, liver steatosis, endotoxin, IL-6 level ↑ *Lactobacillus* and *Bifidobacterium* content.	Probiotic supplementation altered the microbiota and inflammatory system in the high-fat-diet fed rat model.	[52]

High-fat-diet-induced obese mice	Soy-based probiotic drink (*B. longum* ATCC15707 and *Enterococcus faecium* CRL183)	1 × 10^8^ CFU per day for 70 days	↓ IL-6, IL-10. ↓ Size of adipocytes, Body weight gain. ↑ *Bifidobacterium* and *Lactobacillus* content.	Probiotic soy drink positively regulated the intestinal microbiota and immune profile of the high-fat-diet induced obese mice model.	[53]

High-fat-diet induced obese rat	Probiotic formula (*L. rhamnosus* LR5, *L. acidophilus* LA1, *S. thermophilus* ST3, *B. longum* BG7, *B. lactis* BL3, and *B. bifidum* BF3)	1 × 10^7^ CFU per day for 8 weeks	↓ Body weight ↑ *Lactobacillus*, *Bacteroidetes*, and *Bifidobacterium* content. ↓ *Firmicutes* Improved the serum level metabolic profile.	Probiotic supplementation diminished the consequences of high-fat-diet induced obesity in the high-fat-diet induced obese rat model.	[54]

↑: Increased; ↓: Reduced; LH: Luteinizing hormone; FSH: Follicle-stimulating hormone; IL-6: Interleukin-6.

## References

[1] Robson A. A. (2009). Preventing Diet induced disease: Bioavailable nutrient-rich, low-energy-dense diets. *Nutrition and Health*.

[2] Bäckhed F., Ding H., Wang T. (2004). The gut microbiota as an environmental factor that regulates fat storage. *Proceedings of the National Acadamy of Sciences of the United States of America*.

[3] Bäckhed F., Ley R. E., Sonnenburg J. L., Peterson D. A., Gordon J. I. (2005). Host-bacterial mutualism in the human intestine. *Science*.

[4] Sonnenburg J. L., Bäckhed F. (2016). Diet-microbiota interactions as moderators of human metabolism. *Nature*.

[5] Sivamaruthi B. S. (2018). A comprehensive review on clinical outcome of probiotic and synbiotic therapy for inflammatory bowel diseases. *Asian Pacific Journal of Tropical Biomedicine*.

[6] Doron S. I., Hibberd P. L., Gorbach S. L. (2008). Probiotics for the prevention of antibiotic-associated diarrhea. *Journal of Clinical Gastroenterology*.

[7] Isolauri E., Rautava S., Salminen S. (2012). Probiotics in the Development and Treatment of Allergic Disease. *Gastroenterology Clinics of North America*.

[8] Tomaro-Duchesneau C., Saha S., Malhotra M. (2014). Effect of orally administered *L. fermentum* NCIMB 5221 on markers of metabolic syndrome: An *in vivo* analysis using ZDF rats. *Applied Microbiology and Biotechnology*.

[9] Dylag K., Hubalewska-Mazgaj M., Surmiak M., Szmyd J., Brzozowski T. (2014). Probiotics in the mechanism of protection against gut inflammation and therapy of gastrointestinal disorders. *Current Pharmaceutical Design*.

[10] Varankovich N. V., Nickerson M. T., Korber D. R. (2015). Probiotic-based strategies for therapeutic and prophylactic use against multiple gastrointestinal diseases. *Frontiers in Microbiology*.

[11] Sivamaruthi B. S., Kesika P., Chaiyasut C. (2018). Probiotic based therapy for atopic dermatitis: Outcomes of clinical studies. *Asian Pacific Journal of Tropical Biomedicine*.

[12] Tiihonen K., Ouwehand A. C., Rautonen N. (2010). Human intestinal microbiota and healthy ageing. *Ageing Research Reviews*.

[13] Sivamaruthi B. S., Kesika P., Chaiyasut C. (2018). A review on anti-aging properties of probiotics. *International Journal of Applied Pharmaceutics*.

[14] Hou Y.-P., He Q.-Q., Ouyang H.-M. (2017). Human gut microbiota associated with obesity in Chinese children and adolescents. *BioMed Research International*.

[15] Ghoorah K., Campbell P., Kent A., Maznyczka A., Kunadian V. (2016). Obesity and cardiovascular outcomes: a review. *European Heart Journal: Acute Cardiovascular Care*.

[16] World Health Organization Obesity and overweight. https://www.who.int/news-room/fact-sheets/detail/obesity-and-overweight.

[17] Yang G., Staercke C. D., Hooper W. C. (2012). The effects of obesity on venous thromboembolism: a review. *Open Journal of Preventive Medicine*.

[18] Turnbaugh P. J., Ley R. E., Mahowald M. A., Magrini V., Mardis E. R., Gordon J. I. (2006). An obesity-associated gut microbiome with increased capacity for energy harvest. *Nature*.

[19] Kobyliak N., Conte C., Cammarota G. (2016). Probiotics in prevention and treatment of obesity: a critical view. *Nutrition & Metabolism*.

[20] Hall K. D., Guo J., Dore M., Chow C. C. (2009). The progressive increase of food waste in America and its environmental impact. *PLoS ONE*.

[21] Church T. S., Thomas D. M., Tudor-Locke C. (2011). Trends over 5 decades in U.S. occupation-related physical activity and their associations with obesity. *PLoS ONE*.

[22] Apovian C. M., Aronne L. J., Bessesen D. H. (2015). Pharmacological management of obesity: an endocrine society clinical practice guideline. *The Journal of Clinical Endocrinology & Metabolism*.

[23] Popkin B. M., Hawkes C. (2016). Sweetening of the global diet, particularly beverages: Patterns, trends, and policy responses. *The Lancet Diabetes & Endocrinology*.

[24] Sharafedtinov K. K., Plotnikova O. A., Alexeeva R. I. (2013). Hypocaloric diet supplemented with probiotic cheese improves body mass index and blood pressure indices of obese hypertensive patients—a randomized double-blind placebo-controlled pilot study. *Nutrition Journal *.

[25] Jung S.-P., Lee K.-M., Kang J.-H. (2013). Effect of *Lactobacillus gasseri* BNR17 on overweight and obese adults: a randomized, double-blind clinical trial. *Korean Journal of Family Medicine*.

[26] Kim J., Yun J. M., Kim M. K., Kwon O., Cho B. (2018). *Lactobacillus gasseri* BNR17 supplementation reduces the visceral fat accumulation and waist circumference in obese adults: a randomized, double-blind, placebo-controlled trial. *Journal of Medicinal Food*.

[27] Kadooka Y., Sato M., Imaizumi K. (2010). Regulation of abdominal adiposity by probiotics (*Lactobacillus gasseri* SBT2055) in adults with obese tendencies in a randomized controlled trial. *European Journal of Clinical Nutrition*.

[28] Kadooka Y., Sato M., Ogawa A. (2013). Effect of *Lactobacillus gasseri* SBT2055 in fermented milk on abdominal adiposity in adults in a randomised controlled trial. *British Journal of Nutrition*.

[29] Rajkumar H., Mahmood N., Kumar M., Varikuti S. R., Challa H. R., Myakala S. P. (2014). Effect of probiotic (VSL#3) and omega-3 on lipid profile, insulin sensitivity, inflammatory markers, and gut colonization in overweight adults: a randomized, controlled trial. *Mediators of Inflammation*.

[30] Lindsay K. L., Kennelly M., Culliton M. (2014). Probiotics in obese pregnancy do not reduce maternal fasting glucose: a double-blind, placebo-controlled, randomized trial (Probiotics in Pregnancy Study). *American Journal of Clinical Nutrition*.

[31] Halkjaer S. I., Nilas L., Carlsen E. M. (2016). Effects of probiotics (Vivomixx®) in obese pregnant women and their newborn: Study protocol for a randomized controlled trial. *Trials*.

[32] Brahe L. K., Le Chatelier E., Prifti E. (2015). Dietary modulation of the gut microbiota - A randomised controlled trial in obese postmenopausal women. *British Journal of Nutrition*.

[33] Szulińska M., Łoniewski I., van Hemert S., Sobieska M., Bogdański P. (2018). Dose-dependent effects of multispecies probiotic supplementation on the lipopolysaccharide (LPS) level and cardiometabolic profile in obese postmenopausal women: a 12-week randomized clinical trial. *Nutrients*.

[34] Minami J., Kondo S., Yanagisawa N. (2015). Oral administration of *Bifidobacterium breve* B-3 modifies metabolic functions in adults with obese tendencies in a randomised controlled trial. *Journal of Nutritional Science*.

[35] Osterberg K. L., Boutagy N. E., McMillan R. P. (2015). Probiotic supplementation attenuates increases in body mass and fat mass during high-fat diet in healthy young adults. *Obesity*.

[36] Stenman L. K., Lehtinen M. J., Meland N. (2016). Probiotic with or without fiber controls body fat mass, associated with serum zonulin, in overweight and obese adults—randomized controlled trial. *EBioMedicine*.

[37] Madjd A., Taylor M. A., Mousavi N. (2016). Comparison of the effect of daily consumption of probiotic compared with low-fat conventional yogurt on weight loss in healthy obese women following an energy-restricted diet: a randomized controlled trial. *American Journal of Clinical Nutrition*.

[38] Famouri F., Shariat Z., Hashemipour M., Keikha M., Kelishadi R. (2017). Effects of probiotics on nonalcoholic fatty liver disease in obese children and adolescents. *Journal of Pediatric Gastroenterology and Nutrition*.

[39] Gomes A. C., de Sousa R. G. M., Botelho P. B., Gomes T. L. N., Prada P. O., Mota J. F. (2017). The additional effects of a probiotic mix on abdominal adiposity and antioxidant Status: a double-blind, randomized trial. *Obesity*.

[40] Kim M., Kim M., Kang M. (2017). Effects of weight loss using supplementation with *Lactobacillus* strains on body fat and medium-chain acylcarnitines in overweight individuals. *Food & Function*.

[41] Mahadzir M. D. A., Shyam S., Barua A., Krishnappa P., Ramamurthy S. (2017). Effect of probiotic microbial cell preparation (MCP) on fasting blood glucose, body weight, waist circumference, and faecal short chain fatty acids among overweight Malaysian adults: A pilot randomised controlled trial of 4 weeks. *Malaysian Journal of Nutrition*.

[42] Sanchez M., Darimont C., Panahi S. (2017). Effects of a diet-based weight-reducing program with probiotic supplementation on satiety efficiency, eating behaviour traits, and psychosocial behaviours in obese individuals. *Nutrients*.

[43] De Lorenzo A., Costacurta M., Merra G. (2017). Can psychobiotics intake modulate psychological profile and body composition of women affected by normal weight obese syndrome and obesity? A double blind randomized clinical trial. *Journal of Translational Medicine*.

[44] Karbaschian Z., Mokhtari Z., Pazouki A. (2018). Probiotic supplementation in morbid obese patients undergoing one anastomosis gastric bypass-mini gastric bypass (OAGB-MGB) surgery: a randomized, double-blind, placebo-controlled, clinical trial. *Obesity Surgery*.

[45] Minami J., Iwabuchi N., Tanaka M. (2018). Effects of *Bifidobacterium breve* B-3 on body fat reductions in pre-obese adults: A randomized, double-blind, placebo-controlled trial. *Bioscience of Microbiota, Food and Health*.

[46] Mohammadi-Sartang M., Bellissimo N., Totosy de Zepetnek J. O. (2018). The effect of daily fortified yogurt consumption on weight loss in adults with metabolic syndrome: A 10-week randomized controlled trial. *Nutrition, Metabolism & Cardiovascular Diseases*.

[47] Pedret A., Valls R. M., Calderón-Pérez L. (2018). Effects of daily consumption of the probiotic *Bifidobacterium animalis* subsp. *lactis* CECT 8145 on anthropometric adiposity biomarkers in abdominally obese subjects: a randomized controlled trial. *International Journal of Obesity*.

[48] Sanchis-Chordà J., del Pulgar E. M., Carrasco-Luna J., Benítez-Páez A., Sanz Y., Codoñer-Franch P. (2018). *Bifidobacterium pseudocatenulatum* CECT 7765 supplementation improves inflammatory status in insulin-resistant obese children. *European Journal of Nutrition*.

[49] Dardmeh F., Alipour H., Gazerani P. (2017). *Lactobacillus rhamnosus* PB01 (DSM 14870) supplementation affects markers of sperm kinematic parameters in a diet-induced obesity mice model. *PLoS ONE*.

[50] Liao A.-H., Jiang C.-B., Li C.-C. (2017). Combining ultrasound and lactobacilli treatment for high-fat-diet-induced obesity in mice. *Journal of Animal Physiology and Animal Nutrition*.

[51] Chen L.-H., Chen Y.-H., Cheng K.-C. (2018). Antiobesity effect of *Lactobacillus reuteri* 263 associated with energy metabolism remodeling of white adipose tissue in high-energy-diet-fed rats. *The Journal of Nutritional Biochemistry*.

[52] Li X., Song Y., Ma X. (2018). *Lactobacillus plantarum* and *Lactobacillus fermentum* alone or in combination regulate intestinal flora composition and systemic immunity to alleviate obesity syndrome in high-fat diet rat. *International Journal of Food Science & Technology*.

[53] Marchesin J. C., Celiberto L. S., Orlando A. B. (2018). A soy-based probiotic drink modulates the microbiota and reduces body weight gain in diet-induced obese mice. *Journal of Functional Foods*.

[54] Shin J.-H., Nam M. H., Lee H. (2018). Amelioration of obesity-related characteristics by a probiotic formulation in a high-fat diet-induced obese rat model. *European Journal of Nutrition*.

[55] van der Klaauw A., Farooqi I. (2015). The hunger genes: pathways to obesity. *Cell*.

[56] Maclean P. S., Higgins J. A., Giles E. D., Sherk V. D., Jackman M. R. (2015). The role for adipose tissue in weight regain after weight loss. *Obesity Reviews*.

[57] Ochner C. N., Tsai A. G., Kushner R. F., Wadden T. A. (2015). Treating obesity seriously: When recommendations for lifestyle change confront biological adaptations. *The Lancet Diabetes & Endocrinology*.

[58] Leibel R. L., Seeley R. J., Darsow T., Berg E. G., Smith S. R., Ratner R. (2015). Biologic responses to weight loss and weight regain: Report from an American diabetes association research symposium. *Diabetes*.

[59] Pigeyre M., Yazdi F. T., Kaur Y., Meyre D. (2016). Recent progress in genetics, epigenetics and metagenomics unveils the pathophysiology of human obesity. *Clinical Science*.

[60] Montague C. T., Farooqi I. S., Whitehead J. P. (1997). Congenital leptin deficiency is associated with severe early-onset obesity in humans. *Nature*.

[61] Jackson R. S., Creemers J. W. M., Ohagi S. (1997). Obesity and impaired prohormone processing associated with mutations in the human prohormone convertase 1 gene. *Nature Genetics*.

[62] Krude H., Biebermann H., Luck W., Horn R., Brabant G., Grüters A. (1998). Severe early-onset obesity, adrenal insufficiency and red hair pigmentation caused by POMC mutations in humans. *Nature Genetics*.

[63] Clément K., Vaisse C., Lahlou N. (1998). A mutation in the human leptin receptor gene causes obesity and pituitary dysfunction. *Nature*.

[64] Gupta V. K., You Y., Gupta V. B., Klistorner A., Graham S. L. (2013). TrkB receptor signalling: implications in neurodegenerative, psychiatric and proliferative disorders. *International Journal of Molecular Sciences*.

[65] Heymsfield S. B., Wadden T. A. (2017). Mechanisms, pathophysiology, and management of obesity. *The New England Journal of Medicine*.

[66] Tchkonia T., Thomou T., Zhu Y. (2013). Mechanisms and metabolic implications of regional differences among fat depots. *Cell Metabolism*.

[67] McCullough A. J. (2004). The clinical features, diagnosis and natural history of nonalcoholic fatty liver disease. *Clinics in Liver Disease*.

[68] Hall J. E., da Silva A. A., do Carmo J. M. (2010). Obesity-induced hypertension: role of sympathetic nervous system, leptin, and melanocortins. *The Journal of Biological Chemistry*.

[69] Hampel H., Abraham N. S., El-Serag H. B. (2005). Meta-analysis: obesity and the risk for gastroesophageal reflux disease and its complications. *Annals of Internal Medicine*.

[70] Goldring M. B., Otero M. (2011). Inflammation in osteoarthritis. *Current Opinion in Rheumatology*.

[71] Ashrafian H., Toma T., Rowland S. P. (2015). Bariatric surgery or non-surgical weight loss for obstructive sleep apnoea? a systematic review and comparison of meta-analyses. *Obesity Surgery*.

[72] Qin J., Li R., Raes J. (2010). A human gut microbial gene catalogue established by metagenomic sequencing. *Nature*.

[73] Collado M. C., Cernada M., Neu J., Pérez-Martínez G., Gormaz M., Vento M. (2015). Factors influencing gastrointestinal tract and microbiota immune interaction in preterm infants. *Pediatric Research*.

[74] Sekirov I., Russell S. L., Antunes L. C., Finlay B. B. (2010). Gut microbiota in health and disease. *Physiological Reviews*.

[75] Zhao L., Shen J. (2010). Whole-body systems approaches for gut microbiota-targeted, preventive healthcare. *Journal of Biotechnology*.

[76] Claus S. P., Tsang T. M., Wang Y. (2008). Systemic multicompartmental effects of the gut microbiome on mouse metabolic phenotypes. *Molecular Systems Biology*.

[77] Swann J. R., Want E. J., Geier F. M. (2011). Systemic gut microbial modulation of bile acid metabolism in host tissue compartments. *Proceedings of the National Acadamy of Sciences of the United States of America*.

[78] Murphy E. F., Cotter P. D., Healy S. (2010). Composition and energy harvesting capacity of the gut microbiota: relationship to diet, obesity and time in mouse models. *Gut*.

[79] Lopez-Legarrea P., Fuller N. R., Zulet M. A., Martinez J. A., Caterson I. D. (2014). The influence of Mediterranean, carbohydrate and high protein diets on gut microbiota composition in the treatment of obesity and associated inflammatory state. *Asia Pacific Journal of Clinical Nutrition*.

[80] Zhou L., Xiao X. (2018). The role of gut microbiota in the effects of maternal obesity during pregnancy on offspring metabolism. *Bioscience Reports*.

[81] Thursby E., Juge N. (2017). Introduction to the human gut microbiota. *Biochemical Journal*.

[82] Castaner O., Goday A., Park Y.-M. (2018). The gut microbiome profile in obesity: a systematic review. *International Journal of Endocrinology*.

[83] Rosenbaum M., Knight R., Leibel R. L. (2015). The gut microbiota in human energy homeostasis and obesity. *Trends in Endocrinology & Metabolism*.

[84] Rajoka M. S. R., Shi J., Mehwish H. M. (2017). Interaction between diet composition and gut microbiota and its impact on gastrointestinal tract health. *Food Science and Human Wellness*.

[85] Jumpertz R., Le D. S., Turnbaugh P. J., Bogardus C., Gordon J. I., Krakoff J. (2011). Energy-balance studies reveal associations between gut microbes, caloric load, and nutrient absorption in humans. *American Journal of Clinical Nutrition*.

[86] Karlsson F., Tremaroli V., Nielsen J., Bäckhed F. (2013). Assessing the human gut microbiota in metabolic diseases. *Diabetes*.

[87] Turnbaugh P. J., Hamady M., Yatsunenko T. (2009). A core gut microbiome in obese and lean twins. *Nature*.

[88] Walters W. A., Xu Z., Knight R. (2014). Meta-analyses of human gut microbes associated with obesity and IBD. *FEBS Letters*.

[89] Kishino S., Takeuchi M., Park S.-B. (2013). Polyunsaturated fatty acid saturation by gut lactic acid bacteria affecting host lipid composition. *Proceedings of the National Acadamy of Sciences of the United States of America*.

[90] Miyamoto J., Mizukure T., Park S.-B. (2015). A gut microbial metabolite of linoleic acid, 10-hydroxy-cis-12-octadecenoic acid, ameliorates intestinal epithelial barrier impairment partially via GPR40-MEK-ERK pathway. *The Journal of Biological Chemistry*.

[91] Blachier F., Mariotti F., Huneau J. F., Tomé D. (2007). Effects of amino acid-derived luminal metabolites on the colonic epithelium and physiopathological consequences. *Amino Acids*.

[92] Postler T. S., Ghosh S. (2017). Understanding the holobiont: how microbial metabolites affect human health and shape the immune system. *Cell Metabolism*.

[93] Macfarlane G. T., Macfarlane S. (2012). Bacteria, colonic fermentation, and gastrointestinal health. *Journal of AOAC International*.

[94] van der Beek C. M., Dejong C. H. C., Troost F. J., Masclee A. A. M., Lenaerts K. (2017). Role of short-chain fatty acids in colonic inflammation, carcinogenesis, and mucosal protection and healing. *Nutrition Reviews*.

[95] Koh A., De Vadder F., Kovatcheva-Datchary P., Bäckhed F. (2016). From dietary fiber to host physiology: short-chain fatty acids as key bacterial metabolites. *Cell*.

[96] Wang H.-B., Wang P.-Y., Wang X., Wan Y.-L., Liu Y.-C. (2012). Butyrate enhances intestinal epithelial barrier function via up-regulation of tight junction protein claudin-1 transcription. *Digestive Diseases and Sciences*.

[97] Tan J., McKenzie C., Potamitis M., Thorburn A. N., Mackay C. R., Macia L. (2014). The role of short-chain fatty acids in health and disease. *Advances in Immunology*.

[98] Huang W., Guo H., Deng X. (2017). Short-chain fatty acids inhibit oxidative stress and inflammation in mesangial cells induced by high glucose and lipopolysaccharide. *Experimental and Clinical Endocrinology & Diabetes*.

[99] Tolhurst G., Heffron H., Lam Y. S. (2012). Short-chain fatty acids stimulate glucagon-like peptide-1 secretion via the G-protein-coupled receptor FFAR2. *Diabetes*.

[100] Li X., Shimizu Y., Kimura I. (2017). Gut microbial metabolite short-chain fatty acids and obesity. *Bioscience of Microbiota, Food and Health*.

[101] Chambers E. S., Viardot A., Psichas A. (2015). Effects of targeted delivery of propionate to the human colon on appetite regulation, body weight maintenance and adiposity in overweight adults. *Gut*.

[102] de Vadder F., Kovatcheva-Datchary P., Goncalves D. (2014). Microbiota-generated metabolites promote metabolic benefits via gut-brain neural circuits. *Cell*.

[103] Jian H., Yimin J., Shifng P. (2016). Butyrate alleviates high fat diet-induced obesity through activation of adiponectin-mediated pathway and stimulation of mitochondrial function in the skeletal muscle of mice. *Oncotarget *.

[104] Bolognini D., Tobin A. B., Milligan G., Moss C. E. (2016). The pharmacology and function of receptors for short-chain fatty acids. *Molecular Pharmacology*.

[105] Wostmann B. S., Larkin C., Moriarty A., Bruckner-Kardoss E. (1983). Dietary intake, energy metabolism, and excretory losses of adult male germfree wistar rats. *Laboratory Animals*.

[106] Bäckhed F., Manchester J. K., Semenkovich C. F., Gordon J. I. (2007). Mechanisms underlying the resistance to diet-induced obesity in germ-free mice. *Proceedings of the National Acadamy of Sciences of the United States of America*.

[107] Rabot S., Membrez M., Bruneau A. (2010). Germ-free C57BL/6J mice are resistant to high-fat-diet-induced insulin resistance and have altered cholesterol metabolism. *The FASEB Journal*.

[108] Delzenne N. M., Cani P. D. (2011). Gut microbiota and the pathogenesis of insulin resistance. *Current Diabetes Reports*.

[109] Musso G., Gambino R., Cassader M. (2011). Interactions between gut microbiota and host metabolism predisposing to obesity and diabetes. *Annual Review of Medicine*.

[110] Ibrügger S., Kristensen M., Mikkelsen M. S., Astrup A. (2012). Flaxseed dietary fiber supplements for suppression of appetite and food intake. *Appetite*.

[111] Guilherme A., Virbasius J. V., Puri V., Czech M. P. (2008). Adipocyte dysfunctions linking obesity to insulin resistance and type 2 diabetes. *Nature Reviews Molecular Cell Biology*.

[112] Manco M., Putignani L., Bottazzo G. F. (2010). Gut microbiota, lipopolysaccharides, and innate immunity in the pathogenesis of obesity and cardiovascular risk. *Endocrine Reviews*.

[113] Caesar R., Tremaroli V., Kovatcheva-Datchary P., Cani P. D., Bäckhed F. (2015). Crosstalk between gut microbiota and dietary lipids aggravates WAT inflammation through TLR signaling. *Cell Metabolism*.

[114] Moreira A. P. B., Alfenas R. C. G. (2012). The influence of endotoxemia on the molecular mechanisms of insulin resistance. *Nutricion Hospitalaria*.

[115] Muccioli G. G., Naslain D., Bäckhed F. (2010). The endocannabinoid system links gut microbiota to adipogenesis. *Molecular Systems Biology*.

[116] Cani P. D., Neyrinck A. M., Fava F. (2007). Selective increases of bifidobacteria in gut microflora improve high-fat-diet-induced diabetes in mice through a mechanism associated with endotoxaemia. *Diabetologia*.

[117] Nicholson J. K., Lindon J. C. (2008). Systems biology: metabonomics. *Nature*.

[118] Knight R., Callewaert C., Marotz C. (2017). The microbiome and human biology. *Annual Review of Genomics and Human Genetics*.

[119] Park S., Bae J.-H. (2015). Probiotics for weight loss: A systematic review and meta-analysis. *Nutrition Research*.

[120] Zhang Q., Wu Y., Fei X. (2016). Effect of probiotics on body weight and body-mass index: a systematic review and meta-analysis of randomized, controlled trials. *International Journal of Food Sciences and Nutrition*.

[121] Borgeraas H., Johnson L. K., Skattebu J., Hertel J. K., Hjelmesæth J. (2018). Effects of probiotics on body weight, body mass index, fat mass and fat percentage in subjects with overweight or obesity: a systematic review and meta-analysis of randomized controlled trials. *Obesity Reviews*.

[122] Tsai Y.-T., Cheng P.-C., Pan T.-M. (2014). Anti-obesity effects of gut microbiota are associated with lactic acid bacteria. *Applied Microbiology and Biotechnology*.

