# Expression of Concern on “A Review on Role of Microbiome in Obesity and Antiobesity Properties of Probiotic Supplements”

**DOI:** 10.1155/2022/9836248

**Published:** 2022-09-13

**Authors:** BioMed Research International

In the article titled “A Review on Role of Microbiome in Obesity and Antiobesity Properties of Probiotic Supplements” [1], Reference [31] referred to a study using Vivomixx®. We have recently been made aware of a concern regarding the described composition of the Vivomixx® product, and the publishers of the study protocol [2] and results [3] have been informed. These articles may be subject to correction.

## References

[1] Sivamaruthi B. S., Kesika P., Suganthy N., Chaiyasut C. (2019). A Review on Role of Microbiome in Obesity and Antiobesity Properties of Probiotic Supplements. *BioMed Research International*.

[2] Halkjaer S. I., Nilas L., Carlsen E. M. (2016). Effects of probiotics (Vivomixx®) in obese pregnant women and their newborn: study protocol for a randomized controlled trial. *Trials*.

[3] Halkjær S. I., de Knegt V. E., Lo B. (2020). Multistrain Probiotic Increases the Gut Microbiota Diversity in Obese Pregnant Women: Results from a Randomized, Double-Blind Placebo-Controlled Study. *Current Developments in Nutrition*.

